# Data sharing policies: share well and you shall be rewarded

**DOI:** 10.1093/synbio/ysab028

**Published:** 2021-09-08

**Authors:** Jean Peccoud

**Affiliations:** Department of Chemical & Biological Engineering, Colorado State University, Fort Collins, CO, USA

**Keywords:** open science, peer-review, open access, scientific publishing, data management

## Abstract

Sharing research data is an integral part of the scientific publishing process. By sharing data, authors enable their readers to use their results in a way that the textual description of the results does not allow by itself. In order to achieve this objective, data should be shared in a way that makes it as easy as possible for readers to import them in computer software where they can be viewed, manipulated and analyzed. Many authors and reviewers seem to misunderstand the purpose of the data sharing policies developed by journals. Rather than being an administrative burden that authors should comply with to get published, the objective of these policies is to help authors maximize the impact of their work by allowing other members of the scientific community to build upon it. Authors and reviewers need to understand the purpose of data sharing policies to assist editors and publishers in their efforts to ensure that every article published complies with them.

## 1. Introduction

One of the founding principles of *Synthetic Biology* was to promote standardization with the goal of encouraging the reuse of previously described devices (1). Reusing existing components is essential to engineer systems of increasing complexity.

The iGEM Registry of Standard Biological Parts epitomized this aspiration to community sharing as many early synthetic biology used parts from the Registry and later contributed new parts used by other community members working on different projects (2). Many would agree that, to some extent, our community and our discipline have crystallized around this community resource.

While sharing biological material is important, it is also challenging because of the logistics of running a large-scale biobanking operation (3). The costs are significant, and the legal framework can be complicated to navigate (4). As the community grew, it became increasingly reliant on resources like Addgene that benefit a larger scientific community (5, 6). In addition, the need for sharing has progressively shifted from a need to share biological material to the need to share data describing this material (7). The democratization of DNA synthesis (8) makes the annotated sequence of a plasmid a much more valuable asset than the physical access to a poorly documented plasmid. Any properly documented plasmid can be synthesized relatively quickly, whereas it can be very difficult to infer the function of a new genetic design from its raw DNA sequence.

In this context, it is difficult not to observe a disconnect between the community sharing aspirations and its track record of data sharing. Articles are published in high-impact journals that lack critical data (9). Few datasets are published in specialized journals like *Scientific Data*. *Synthetic Biology* has never received a single dataset submission, even though it has been accepting this type of submission for several years.

Considerable efforts have been dedicated to the development of data exchange standards [10–12]. Dedicated data sharing resources have been available to the community (13), but they appear to be somewhat underutilized.

When *Synthetic Biology* was started, a lot of attention was given to the data and material sharing policies (14). These policies were revised several times to make them easier to understand. Checklists were provided to help authors and reviewers evaluate this critical aspect of submissions.

Yet, despite all these efforts, it is still difficult to enforce these policies. It seems that many authors and reviewers do not completely understand their purpose.

## 2. Rationale for sharing data

The purpose of sharing data is not to please the editor. It is not an administrative requirement that journals impose on their author as a rite of passage. It is not a requirement for political correctness or some courtesy necessary to be a good member of the scientific community.

Authors are asked to share data so that their readers can use the data described in their publications. The ability of a reader to use the results described in an article often depends on the availability of key data.

For example, articles describing sets of plasmids without providing the plasmid sequence force the reader to reverse engineer the plasmid sequences based on the article content. This is time consuming at best and often not even possible. In many cases, the data are more important than the narrative describing the data. For example, the description of how a plasmid was assembled by combining restriction fragments from different origins is no longer useful nor sufficient.

It is not sufficient because it does not make it possible to reconstruct the plasmid sequence if the sequence of the original plasmids is not available. It is not useful because the way a plasmid was produced is largely irrelevant today. The final sequence and its annotations are what make it possible for readers to understand the article and reuse its results in their own research.

When selecting what data to share, authors need to think as readers. They should wonder what data they would need if they were reading their article. Properly annotated DNA sequences are something that most synthetic biology papers should provide. Any kind of functional testing results is also desirable, especially when they could lead to different analyses or interpretations or could be included in reproducibility studies (15). Increasingly, software scripts used to analyze the data are becoming an important element of data sharing as they are essential to ensure the reproducibility of computational workflows (16).

It is also important to share intermediate data like DNA sequencing reads that may not necessarily be reused but would give reviewers and authors confidence that the authors have properly validated the physical sequences of the plasmids and organisms they describe.

Authors should remember that they share data because it benefits them. Between two equally interesting papers, the one that was easier to build upon because it properly shared data is the one most likely to get cited (17). Similarly, data demonstrating that all the controls were properly performed will give the article more authority. Sharing data is an integral part of the publication process and contributes to publication quality.

## 3. Ways to share data

The way data is shared is just as important as the nature of the data that are shared. Here, again, authors should think like readers and wonder how they would want to receive the data associated with their manuscript.

### 3.1 Format

The file format needs to be adequate for the data. For example, a PDF file is not the proper way to share annotated DNA sequences. Some readers may be able to recover a GenBank file embedded in a PDF file, but nobody should be asked to do that. Similarly, including a GenBank file in a large Word document is equally problematic because it would require a lot of file manipulation to extract the sequence information from the Word document and translate it into a format that can be recognized by a bioinformatics software application. Annotated plasmid sequences should be provided as GenBank files or other standard file formats compatible with most plasmid editors.

Similarly, plasmid sequences should not be provided as text files or FASTA files because these formats do not capture annotations, an essential component of engineered sequences.

However, FASTA files or Excel spreadsheets would be suitable formats to share primer sequences or any other set of sequences that do not need annotations. Again, including these sequences in Word documents or PDF files is not doing much to help the reader reuse these sequences.

Similarly, most phenotypic data can be shared as Excel spreadsheets or delimited text files that can be easily imported into data analysis software applications. Including them in Word documents is not particularly helpful.

Word documents and PDF files can be great to share additional methods and figures that do not fit in the main manuscript. They are never the right formats to share data.

### 3.2 Depositing shared data

Authors have several options to deposit the data they want to share.

Journals like *Synthetic Biology* allow authors to upload data associated with their manuscript. These files are often referred to as online supplement or supplementary data. While this option is convenient, it has limitations. The amount of data that can be uploaded might exceed the size of the datasets that need to be shared. The second limitation is the impossibility of updating the dataset postsubmission. The supplementary data of an article are frozen at the time of the submission and cannot be easily modified after the manuscript is submitted. Finally, supplementary data submitted to the journal do not adhere to data formats defined by the scientific community.

The FAIRSharing project (18) provides a manually curated database of data repositories. This resource provides pointers to community-driven resources to properly share virtually any kind of scientific data. Authors are encouraged to deposit their data with the appropriate repository and cite their submission in their manuscript rather than including data in their manuscript online supply.

Another option consists in using a nonspecialized data repository like Dryad or Figshare. These websites provide large amounts of cloud storage that can host any kind of data. They allow data submissions to be discoverable and citable by issuing them a Digital Object Identifier. Data submissions can be regularly updated at any time without losing the previously published versions of the datasets. That makes it possible to add data while the manuscript is under review or after it was published.

A good data sharing strategy may involve depositing different data in different locations or possibly the same data in different formats and different locations for maximum visibility.

### 3.3 Timing of data sharing

When using the journal online supplement to share data, the data are published at the same time as the article they are associated with. However, this traditional approach is not the only one to consider.

Over the last 20 years, the National Institutes of Health has progressively refined its data sharing policies mostly as a response to the needs of the genomics community (19). This is a field where data generation and data analysis are two very clearly distinct aspects of research projects that are not necessarily completed by the same people. Because data analysis can be complex and slow, people generating data have been encouraged to release the data they produced before they completed their analysis. This created opportunities for other members of the scientific community to perform their own analyses of these valuable datasets. The same argument can apply to any project. The results of many research projects are often published well after the data were produced. Depositing the data as they are produced creates opportunities for other scientists to use the data in the same way as the publication of a preprint makes the results of a project available to the community before the manuscript completes the peer-review process. Nonspecialized repositories like Figshare make it very easy to publish data as they are produced and before they can be submitted to a journal and to specialized repositories.

Postpublication data publication is also worth considering. Properly documenting a dataset can be time consuming. When trying to publish important results, resources are often allocated to producing data and writing the manuscript. Limited bandwidth may be available for data packaging, and the data shared upon the manuscript submission may not always be organized to maximize reuse by others. After the main manuscript has been submitted or published, authors can consider submitting another paper describing a more polished version of their data. Dataset is one submission type offered by *Synthetic Biology*.

## 4. Conclusion

*Synthetic Biology* has developed data sharing policies with the goal of helping authors maximize the impact of the work they submit for publication to the journal. Authors and reviewers need to understand the goals of these policies to help enforce them and contribute to their evolution.
